# Malignant pleural mesothelioma in Italy

**DOI:** 10.4103/0019-5278.55124

**Published:** 2009-08

**Authors:** Claudio Bianchi, Tommaso Bianchi

**Affiliations:** Center for the Study of Environmental Cancer, Italian League against Cancer, Hospital of Monfalcone, 34074 Monfalcone, Italy

**Keywords:** Asbestos, latency periods, mesothelioma, necropsy, pleura, shipbuilding

## Abstract

This study reviews a series of 811 malignant pleural mesothelioma cases, diagnosed at hospitals in Trieste and Monfalcone districts of north eastern Italy, a narrow coastal strip with a population of about three lakh, in the period 1968-2008. The diagnosis was based on histological examination in 801 cases, and cytological findings in 10. Necropsy was performed in 610 cases. Occupational histories were obtained directly from the patients or their relatives through personal or telephone interviews. Routine lung sections were examined for asbestos bodies in 500 cases. In 143 cases asbestos bodies were isolated and counted by chemical digestion of the lung tissue using the Smith-Naylor method. The series included 717 men and 94 women aged between 32 and 93 years (mean 69.2 years). Detailed occupational data was obtained for 732 cases.

The majority of patients had marine jobs - shipbuilding (449 cases), maritime trades (56 cases), and port activities (39 cases). The nature of work of other patients included a variety of occupations, with non-shipbuilding industries being the most common. Thirty-four women cleaned the work clothes of family members occupationally exposed and hence had a history of asbestos exposure at home. Most of the patients had their first exposure to asbestos before 1960. The latency period ranged between 13 and 73 years (mean 48.2). Latency period among insulators and dock workers were shorter than other categories. Asbestos bodies were detected on routine lung sections in 343 cases (68.6%). Lung asbestos body burdens after isolation ranged between two to 10 millions bodies per gram of dried tissue. Despite some limitations in the use of asbestos in this area since the 1970s, the incidence of tumor remained high during the last years.

## INTRODUCTION

In the past decades the incidence of malignant mesothelioma has markedly increased in various countries.[1] The geographic distribution of mesothelioma shows that the incidence rates are highest in areas with high shipbuilding activity or with intense asbestos-cement production.[1,2] At a national level, data points to a relationship between mesothelioma mortality and asbestos consumption some decades ago.[3–5]

Studies which began in 1971 showed a high incidence of mesothelioma in the Trieste-Monfalcone area, on the border with Slovenia and large shipyards of the region were identified as the main source of mesothelioma epidemic.[6–11] This investigation has been conducted to delineate the principal features of pleural mesothelioma in the above area over a long period.

## MATERIALS AND METHODS

The cases included in the study were diagnosed at hospitals of Trieste in the period 1968-2008 and at the hospital of Monfalcone in the period Oct 1979-2008. Pathological diagnosis was based on histological examinations in 801 cases and cytological findings in 10 cases. In 610 cases the diagnosis was confirmed or performed at necropsy.

Occupational data was collected directly from the patients or their relatives through personal or telephone interviews. In addition, enquiries were conducted to reconstruct the characteristics of exposure to asbestos in the area during the past decades. For this purpose, a number of people employed in different branches in the past in Trieste and Monfalcone were interviewed.

Routine lung sections were examined for asbestos bodies in 500 cases. Isolation and quantization of asbestos bodies were performed in 143 cases, after chemical digestion of lung tissue, using the Smith-Naylor method.[12]

## RESULTS

The study included 717 men, aged between 32 and 93 years (mean 68.7, median 69.0), and 94 women aged between 40 and 93 years (mean 73.2, median 73.0) [Table 1]. The patients had worked in a variety of workplaces, most frequently in shipyards [Table 2]. In 79 cases (57 men and 22 women), occupational data was insufficient. Thirty-four women reported history of domestic exposure to asbestos. They had cleaned the work clothes of their family members who had been occupationally exposed. In cases classified as ‘other,’ the patients had been employed in a wide range of branches including railways, construction industry, pastry and bakery, army and police, municipal water department. Unusual occupations included telephone technician, movie projectionist, mattress maker, auto mechanic, fireman, asbestos company agent, etc. Environmental exposure to asbestos was retained as the cause of mesothelioma in two cases, and suspected in two. In one case the patient had been treated with radiotherapy for Hodgkin's disease, 34 years ago.

**Table 1:** Sex and age distribution in 811 cases of malignant pleural mesothelioma, Trieste-Monfalcone area, 1968-2008

Age groups (years)	No. of men	%	No. of women	%	Total	%
30-39	2	0.3	0	0.0	2	0.2
40-49	17	2.4	4	4.3	21	2.6
50-59	116	16.2	8	8.5	124	15.3
60-69	235	32.8	22	23.4	257	31.7
70-79	236	32.9	42	44.7	278	34.3
80-89	104	14.5	17	18.1	121	14.9
90-99	7	1.0	1	1.1	8	1.0
Total	717	100.0	94	100.0	811	100.0

**Table 2:** Occupational data in 732 cases of malignant pleural mesothelioma, Trieste-Monfalcone area, 1968-2008

Exposure type	No. of men	%	No. of women	%	Total	%
Insulation	13	2.0	0	0.0	13	1.8
Shipbuilding	440	66.7	9	12.5	449	61.3
Maritime trades	56	8.5	0	0.0	56	7.7
Port activities	39	5.9	0	0.0	39	5.3
Other industries*	57	8.6	16	22.2	73	10.0
Domestic exposure	0	0.0	34	47.2	34	4.6
Other	55	8.3	13	18.1	68	9.3
Total	660	100.0	72	100.0	732	100.0

* Non-shipbuilding machinery, non-asbestos textile, petrochemical, iron work, etc.

About 80% of the patients had their first exposure before 1960 [Table 3]. The duration of exposure to asbestos was calculated in 504 cases; 73% had been exposed for 20 years or more [Table 4]. The latency periods, defined as time intervals elapsed between first exposure to asbestos and diagnosis of mesothelioma, were calculated in 552 cases; in 77% of cases latency periods were longer than 40 years [Table 5]. Latency periods varied markedly from an occupational group to another [Table 6].

**Table 3:** First exposure to asbestos in 587 cases of malignant pleural mesothelioma, Trieste-Monfalcone area, 1968-2008

Calendar years	No. of cases	%
1910-19	8	1.4
1920-29	74	12.6
1930-39	153	26.1
1940-49	131	22.3
1950-59	109	18.6
1960-69	87	14.8
1970-79	25	4.3

**Table 4:** Duration of exposure to asbestos in 504 cases of malignant pleural mesothelioma, Trieste-Monfalcone area, 1968-2008

Duration (years)	No. of cases	%
0-9	81	16.1
10-.19	54	10.7
20-29	100	19.8
30-39	162	32.1
40-49	104	20.6
50-59	3	0.6

**Table 5:** Latency periods in 552 cases of malignant pleural mesothelioma, Trieste-Monfalcone area, 1968-2008

Years	No. of cases	%
10-19	1	0.2
20-29	29	5.3
30-39	98	17.8
40-49	144	26.1
50-59	187	33.9
60-69	83	15.0
70-79	10	1.8

**Table 6:** Latency periods in 552 cases of malignant pleural mesothelioma by category of exposure, Trieste-Monfalcone area, 1968-2008

Category	No. of cases	Range	Mean	St. dev.	Median
Insulation	11	27-49	34.8	8.3	33.0
Shipbuilding	378	13-73	48.7	10.9	51.0
Maritime trades	43	35-71	55.3	8.4	56.0
Port activities	28	25-60	37.1	12.1	33.0
Other industries	49	28-69	46.0	9.9	47.5
Domestic exposure	15	27-62	50.8	10.9	55.0
Other	28	25-64	44.9	9.2	46.0

Asbestos bodies were detected on routine lung sections in 343 cases (68.6%). The burden of asbestos bodies after isolation ranged from two bodies to about 10 million per gram of dry tissue [Table 7].

**Table 7:** Lung asbestos body counts in 143 malignant pleural mesotheliomas, Trieste-Monfalcone area, 1968-2008

No. of asbestos bodies	No. of cases
0-999	31
1,000-9,999	51
10,000-99,999	40
100,000-999,999	19
> 1,000,000	2

## DISCUSSION

A singular feature of this mesothelioma series is the high proportion of cases of diagnosis confirmed at necropsy. This offers a more reliable basis to the findings of the study. Although mesothelioma has been investigated in depth during the last decades, the diagnosis of this tumor often remains difficult.[13,14] In addition, necropsy furnishes precious information about asbestos exposure the person had. This is particularly relevant since the occupational history of a patient with mesothelioma is sometimes difficult to interpret. Given the long latency periods of the tumor, occupational data regards even the remote past. Sometimes the factory in which the patient had worked has ceased its activity since decades; the reconstruction of such distant work conditions becomes a question of industrial archaeology.

In the Trieste-Monfalcone area, pleural mesothelioma is a condition mainly affecting men. Regarding age, 96% of the patients were between 50 and 89 years, and 66% between 60 and 79.

Working in shipyards emerged as the occupation for most people with pleural mesothelioma in the study area. Numerous studies conducted in Europe, USA and Japan point to the strong relationship between shipbuilding and mesothelioma.[10,15–25] The high incidence of mesothelioma among shipyard workers is considered the effect of asbestos exposure occurred in the shipyards. The relevance of such an exposure has been documented by several studies.[25,26] As far as north eastern Italy is concerned, a series of investigations carried out at Monfalcone Hospital in the period October 1979-September 1998 explored markers of asbestos exposure (pleural plaques, lung asbestos bodies) in a series of 3,640 consecutive necropsies.[26] Lifetime occupational data for 1,277 patients was obtained. The shipyard workers were characterized by very high prevalence of pleural plaques (86.7%), high prevalence of asbestos bodies on routine lung sections (35.3%), and high amounts of lung asbestos bodies after isolation. The data gathered indicates that, asbestos exposure was an ubiquitous and heavy occurrence in shipyards of the area.

Mesothelioma was first identified among shipyard workers in Trieste in 1972-73.[6,7] However, a retrospective examination of necropsy records carried out at the Trieste Hospital in the period 1944-1966 revealed some cases diagnosed in 1940s and 1950s.[27] In this context it has to be remembered that asbestos-related mesothelioma has been observed in various countries since 1936.[28]

Maritime trade is an occupational branch encountered in various cases of the present mesothelioma series. As a consequence of large asbestos use in shipbuilding seamen are at risk for mesothelioma.[29,30] Seafarers in merchant marine as well as people employed in the navy are at risk.[29] A series of analyses of board maritime shipping in the US, in the period 1978-1992, revealed extremely low concentrations of asbestos fibers.[30] However, this fact does not efface the bulk of epidemiological evidence indicating a link between maritime trade and mesothelioma.

Increase in number of pleural mesothelioma cases was recently observed in Trieste among port workers,[31] with patients generally employed in loading-unloading of a variety of goods, including asbestos. In the period 1960-1998, over five lakh tons of asbestos passed through the port of Trieste[32] by sea traffic (arrivals plus departures) ranging between 558 and 18,882 tons per year in the period 1960-1996. Until late 1970s, and partly even in 1980s, asbestos passing through the Trieste port was transported using jute or paper sacks. Such sacks often broke, resulting in high dustiness. Enquiries by the Occupational Medicine Unit of the Local Health Authority, in 1977, documented the severity of the pollution at Trieste port.

Seventy-three people had worked in various industries, including non-shipbuilding machinery, non-asbestos textile, petrochemical, iron work, etc. Thirty-four women had been exposed to asbestos at home. They had cleaned the work clothes of their family members employed in workplaces polluted by asbestos, mainly the shipyards.

The patients, included in the category ‘other’, represented a heterogeneous group of particular interest. In fact, many of these occupations have been underestimated in the past as possible sources of asbestos exposure and other remain unrecognized as work at risk for asbestos disease. Working as telephone technician,[33] or as movie projectionist are examples of such scarcely considered occupations.

About 80% of the patients in the present series had their first exposure before 1960. In nearly three-fourth of the cases, the duration of exposure was 20 years or more; half the patients, for whom sufficiently detailed data were available, had been exposed for 30-40 years or more. This point deserves attention. It is generally strongly emphasized that mesothelioma may develop after very short exposures. If this is true, it is also true that majority of the people with mesothelioma have a history of long exposure.

As far as latency periods are concerned, the present findings differ markedly from those generally reported in literature. We found latency periods of 40 years or more in three-fourth of the cases although 20-40 years were mostly quoted. Similar findings have also been observed in other series.[34] Data obtained (including data from present study) suggests an inverse relationship between intensity of exposure to asbestos and length of the latency periods.[34–37] Longer latency period is seen after environmental exposure than after occupational exposure,[38,39] supporting the above idea. The intensity of exposure is generally lower in the former than in the latter.

In the study area, limitations in use of asbestos began in the second half 1970s. A further reduction occurred in the shipyards of the area in 1980s. A total ban was imposed in Italy in 1992. This means that during the last three decades, the global dose workers had been lower in comparison with previous decades. Nevertheless, mesothelioma epidemic did not show signs of abatement during the last years.[40–42] Mesothelioma continues to represent a major problem in the area. Screening of mesothelioma among asbestos exposed people has recently been tried in Monfalcone,[43] but the results have not been encouraging.
